# Anesthetic Management for Lower Limb Fracture in Severe Aortic Valve Stenosis and Fat Embolism: A Case Report and Review of Literature

**DOI:** 10.5812/aapm.13713

**Published:** 2014-03-08

**Authors:** Faranak Rokhtabnak, Mohammad Mahdi Zamani, Alireza Kholdebarin, Alireza Pournajafian, Mohammad Reza Ghodraty

**Affiliations:** 1 Department of Anesthesiology and Pain Medicine, Firoozgar Hospital, Iran University of Medical Sciences, Tehran, Iran

**Keywords:** Aortic Valve Stenosis, Fat Embolism, Sciatic Nerve, Femoral Nerve

## Abstract

**Introduction:**

Anesthesia in severe aortic stenosis, which describes a valve surface area less than 1 cm^2^, can result in rapid clinical deterioration and patient mortality. These patients may require treatment for aortic stenosis before any surgical intervention. In suitable patients percutaneous balloon aortic valvutomy appears to carry lower risk, but in emergency situations, it is important to determine which kind of anesthesia technique has the lowest risk for these patients, without any cardiac intervention.

**Case Presentation:**

In this case report, we present a patient who had tibia and fibula fractures and a symptomatic severe critical aortic stenosis which was diagnosed during a preoperative visit. The patient had exertional dyspnea, palpitations and fainting history, but he had not received any medical therapy before the present admission. During hospitalization and preoperative evaluation, a fat embolism occurred and the patient was admitted to the intensive care unit. Immediately after his recovery, we successfully managed the tibia and fibula fracture fixation without any cardiac intervention.

**Conclusions:**

Our anesthesia method was sciatic and femoral nerve block under double ultrasonic and nerve stimulator guidance.

## 1. Introduction

Despite impressive advances in anesthesia and surgical techniques, the morbidity and mortality of patients with severe aortic stenosis (AS) remains high (1). According to previous studies, the severity of AS is highly predictive of complications (2). Mild to moderate AS is well tolerated during anesthesia, but in severe AS the hemodynamic parameters can rapidly deteriorate and this is a risk factor for perioperative mortality and morbidity. Therefore, aortic valve replacement (AVR) improves survival before any other surgery (3). On the other hand, orthopedic surgeries are the most commonly reported non-cardiac surgeries among patients with severe AS (4) and fat embolism syndrome (FES) are accompanied by an incidence of less than 1% of long bone fractures (5). To the best of our knowledge there have not been any reports of anesthesia experiences for patients with combined FES and severe AS, in the literature. Therefore, we report a case of a patient with successful anesthetic management of severe AS with a fat embolism, who underwent tibia and fibula nailing surgery.

## 2. Case Presentation

The authors obtained the patient's consent and consulted the Institutional Ethics Review Board (IRB) for approval (not deemed necessary by the IRB) for publishing in this journal. A 18 year old man, weight 50 kg, height 173 cm, was admitted in our center, with tibia and fibula fractures and undiagnosed severe congenital AS, he presented in CHF with functional capacity II (New York Heart Association Functional Classification). Preoperative examination attained findings revealed his valvular heart disease. He had palpitations and a fainting history, but had not receive any medical therapy before the present admission. An electrocardiogram (ECG) showed left ventricular hypertrophy (LVH) and strain pattern. Echocardiography revealed a bicuspid aortic valve, thickened leaflets, aortic valve area of 0.75 cm^2^, pressure gradient across aortic valve (peak) of 100 mm Hg, concentric LVH, and ejection fraction of 60%. Cardiologic consult reported high risk surgery for this patient, but because of a partially good functional capacity and preserved EF, they did not recommend AVR before orthopedic surgery. On the second day of admission, acute signs and symptoms of FES were observed in the patient. He had hypoxemia (SPO_2 _= 83%), tachypnea (34/min), tachycardia (120-140 beats/min), acute mental status deterioration, petechiae over the chest and in conjunctiva, thrombocytopenia, and anemia (Hb: 8.1 g/dL). Blood gas (ABG) analysis showed hypoxemia (PaO_2_ of 57.6 mm Hg), and metabolic acidosis (PH 7.27, base excess of -5.8). The patient was intubated and transferred to the intensive care unit (ICU). Mechanical ventilation with FIO_2_ of 1.0, IV methyl-prednisolone (125 mg/qid), infusion of heparin (18 units/kg/h), and delay in orthopedic surgery until symptoms recovery, were administrated. Fortunately he recovered after about 13 hours and was extubated by himself on the 3rd day of admission. After two units of packed RBC transfusion, his Hb rose to 10.8 g/dL, he was then sent to the operating room on the morning of the 4th day. The patient received all cardiac medications, oxazepam (10 mg) and ranitidine (50 mg), one hour before surgery, and anticoagulation was on hold for 6 hours before surgery. In the operation room, he had stable vital signs and his ABG analysis was normal. The last chest X-ray showed cardiomegaly, and an ECG revealed sinus rhythm and LVH. He received 2 mg midazolam, 100 µg fentanyl for premedication, and he was then put on monitoring (ECG, noninvasive blood pressure, pulse oximetry and urine output) and oxygen therapy with a mask. For management of the anesthesia, after informed consent was obtained, a sciatic and femoral nerve block (SFB), with ultrasound and nerve stimulator guided method (double guidance), was selected. SFB was performed, using a nerve stimulator (Temena, Felsberg-Gensungen, Germany) delivering 0.5 mA impulse for femoral nerve and 1 mA for sciatic nerve (0.1 ms) at 1 Hz and a SonoSite S-Nerve ultrasound platform.

An 8 to 15 MHz linear transducer (SonoSite, Bothell, Washington) was used for femoral nerve block only. Femoral nerve block was done by a 22 gauge, 50 mm short beveled, Teflon-coated needle (Locoplex, Vygon, Ecouen, France), with a 20 mL lidocaine 1% and bupivacaine 0.25% combination, and when nerve stimulation was led to a contraction of the quadriceps muscle and anterior movement of the patella, 20 cc of local anesthetic was injected. T sciatic nerve block was done by an anterior approach, with a 22 gauge, 150-mm short beveled, Teflon-coated needle (Locoplex, Vygon, Ecouen, France) needle and and when nerve stimulation was led to any foot movement, 25 mL of the previously mentioned local anesthetic drug combination. In case of failure in our regional techniques, our rescue method was general anesthesia (GA) with appropriate drugs (etomidate, cisatracuriom plus opioids and nitrose oxide) which may be anesthesiologists preferred choice for patients suffering from serious cardiac complications, including severe AS, or if their peripheral nerve block fails before or during surgery. 

Surgery was started after twenty minutes, during which the patient was comfortable and did not sense any pain; furthermore, there were no hemodynamic complications during the next 150 minutes of operation time. Further course of recovery was uneventful, and the patient was discharged from the hospital in a satisfactory condition on the 6th day of admission. After six months, follow up was performed by phone and no orthopedic or general condition complications were reported.

## 3. Discussion

We report a successful utilization of combined SFB for open reduction and internal fixation (ORIF) of a leg fracture in a young patient who had severe AS, complicated with a fat embolism. General anesthesia (GA) could have been an option in our patient. Although, GA can decrease myocardial activity, produce vasodilatation, and finally create wide changes in hemodynamic variables. Regional anesthesia is widely used for the anesthetic management of surgical repair of lower limb fractures. These consist of spinal anesthesia (SA) and regional nerve blocks which include, femoral nerve block, sciatic nerve block, SFB, triple nerve block (femoral, obturator and sciatic nerves), psoas (lumbar plexus) block, or continuous epidural block (6). But in cases with severe AS, large decreases in systemic vascular resistance (SVR) should be avoided. There have been reports of hypotension-induced ischemia which has presented in patients with left ventricular outflow tract obstruction, who received SA (7). However, when regional anesthesia is chosen, epidural is recommended rather than SA, because of the gradual onset of the peripheral sympathetic nervous system blockade (8).

A meta-analysis on hip fracture surgery, comparing GA with regional anesthesia concluded that postoperative deep venous thrombosis is less common with regional anesthesia. This meta-analysis reported a less than 1% hip fracture operative mortality, of which pulmonary embolism is the most common cause of death (9). There have also been reports of a lower incidence of myocardial infarction in patients who had received regional anesthesia compared with those who received GA (10); moreover, a regional anesthesia means central neuroaxial blocks, which induce vasodilatation below the level of the block, resulting in large decreases in SVR and consequently hypotension, that may have negative implications for patients with severe AS (11). There are no contraindications for the use of an epidural block in patients with severe AS, and there have been reports of its successful use. On the other hand, an epidural block may cause a sharp decline in SVR due to sympathetic block, and this can result in systemic hypotension and reduced coronary perfusion in these patients, which is not recommended in severe AS. Furthermore, according to a meta-analysis, using this technique is better carried out as a continuous epidural catheter infusion (11).

There is little evidence on the advantages of continuous SA. Several studies have reported improved hemodynamic control with continuous SA over epidural or single-dose SA in healthy patients (12-13). Collard et al. utilized continuous SA in two patients with severe AS undergoing surgery on the lower extremities. The authors suggested invasive hemodynamic monitoring using a PA catheter, and this could enable assessment of systemic blood pressure, pulmonary artery pressure (PAP), SVR and cardiac output (CO) for injecting enough intratechal local anesthetic and also for adequate hydration (14).

Regional anesthesia, such as SFB, which blocks the peripheral nerves, produces good anesthesia and that is an effective regional technique for fixation of tibia and fibula fractures; due to the unilateral nature of this block, less change in SVR occurs, compared to central neuraxial blocks. Three approaches of sciatic nerve block are; anterior, posterior and lithotomy; the anterior approach is the modern approach and this has several advantages over the others, as while both sciatic and femoral blocks can be performed with the patient in the same position (in supine position), the anterior approach using surface anatomical landmarks has been associated with technical difficulties (15), due to the location of the sciatic nerve which varies among individuals (16). Ota et al. compared the anterior and posterior approach to sciatic nerve block under real-time ultrasound guidance, and reported that the anterior technique can be performed easily and successfully for minor knee surgery, under real-time ultrasound guidance (16).

Lumbar plexus combined with parasacral sciatic nerve block has also been reported for anesthesia of hip surgery in a patient with severe AS (17), but there are disadvantages of using these techniques. They are more difficult techniques, more time consuming, require larger volumes of local anesthetic and result in more failures compared with SFB.

Our case developed fat embolism syndrome (FES) simultaneously with his long bone fracture. FES typically appears 12 to 72 hours after long-bone fractures, especially in fractures of the femur or tibia. The triad of; hypoxemia, mental confusion, and petechiae, in patients with fractures should arouse suspicion of a fat embolism. Associated pulmonary dysfunction may be limited to arterial hypoxemia, which is always present, or it may progress from tachypnea to acute respiratory distress syndrome (18). Central nervous system dysfunction ranges from confusion to seizures and coma. Petechiae, especially over the neck, shoulders, and chest, occur in at least 50% of patients (18). Bulger et al. have shown that early intramedullary fixation does not increase the incidence or severity of FES (19). Therefore, we opted to perform ORIF as soon as possible after recovery from the first FES episode, in order to reduce infection of the fracture site and lower the resulting cardiovascular adverse effects of sepsis in this particular case. We used ultrasound and nerve stimulation to locate both nerves accurately, and so relatively small volumes of local anesthetic were administered in view of the patient’s clinical condition.

In summary, this case report presents a successful case of tibia and fibula fracture fixation without any cardiac intervention in a patient with severe AS and simultaneous FES, describing our preference for SFB in a patient with a notably complex clinical picture. Moreover, to our knowledge, this is the first experience of such a case reported in the literature.
